# Differences in Emotional and Pain-Related Language in Tweets About Dentists and Medical Doctors: Text Analysis of Twitter Content

**DOI:** 10.2196/10432

**Published:** 2019-02-06

**Authors:** Jan-Are K Johnsen, Trude B Eggesvik, Thea H Rørvik, Miriam W Hanssen, Rolf Wynn, Per Egil Kummervold

**Affiliations:** 1 Department of Clinical Dentistry Faculty of Health Sciences UiT The Arctic University of Norway Tromsø Norway; 2 Department of Clinical Medicine Faculty of Health Sciences UiT The Arctic University of Norway Tromsø Norway; 3 Norut Northern Research Institute Tromsø Norway

**Keywords:** dental anxiety, dentistry, psychology, social media, internet, dental public health, Twitter, professional role, occupational stereotype

## Abstract

**Background:**

Social media provides people with easy ways to communicate their attitudes and feelings to a wide audience. Many people, unfortunately, have negative associations and feelings about dental treatment due to former painful experiences. Previous research indicates that there might be a pervasive and negative occupational stereotype related to dentists and that this stereotype is expressed in many different venues, including movies and literature.

**Objective:**

This study investigates the language used in relation to dentists and medical doctors on the social media platform Twitter. The purpose is to compare the professions in terms of the use of emotional and pain-related words, which might underlie and reflect the pervasive negative stereotype identified in relation to dentists. We hypothesized that (A) tweets about dentists will have more negative emotion-related words than those about medical doctors and (B) pain-related words occur more frequently in tweets about dentists than in those about medical doctors.

**Methods:**

Twitter content (“tweets”) about dentists and medical doctors was collected using the Twitter application program interface 140Dev over a 4-week period in 2015, scanning the search terms “dentist” and “doctor”. Word content of the selected tweets was analyzed using Linguistic Inquiry and Word Count software. The research hypotheses were investigated using nonparametric Wilcoxon-Mann-Whitney tests.

**Results:**

Over 2.3 million tweets were collected in total, of which about one-third contained the word “dentist” and about two-thirds contained the word “doctor.” Hypothesis A was supported since a higher proportion of negative words was used in tweets about dentists than in those about medical doctors (*z*=−10.47; *P*<.001). Similarly, tests showed a difference in the proportions of anger words (*z*=−12.54; *P*<.001), anxiety words (z=−6.96; *P*<.001), and sadness words (*z*=−9.58; *P*<.001), with higher proportions of these words in tweets about dentists than in those about doctors. Also, Hypothesis B was supported since a higher proportion of pain-related words was used in tweets about dentists than in those about doctors (*z*=−8.02; *P*<.001).

**Conclusions:**

The results from this study suggest that stereotypes regarding dentists and dental treatment are spread through social media such as Twitter and that social media also might represent an avenue for improving messaging and disseminating more positive attitudes toward dentists and dental treatment.

## Introduction

An increasing number of people use social media, such as Facebook and Twitter; these are becoming central as news outlets and are even creating headline news themselves, for example, when the US President tweets and stirs controversy [1]. The ubiquity of social media has created an opportunity for researchers to use these tools as sources of data on a range of topics, including on the spread of illnesses and attitudes to health-related topics [2-4]. A few studies have also used social media to examine public opinion on dental health, especially fluoridation [5,6]. However, prior research has suggested that social media may be used to spread distorted or false information and that such information may have important negative consequences, for instance, when dangerous information is spread about how to contain epidemics or when mental disorders are associated with negative emotions and unsupportive tweets [7,8].

With new technologies and social media, there are new venues for health communication, as well as new venues for expressing stereotypes and social categories. In order to make sense of the world, people have a tendency to think about others in terms of stereotypes or categories [9,10], and the existence of occupational stereotypes is relatively well established [11,12]. This tendency has benefits for saving cognitive resources [13] but can pose problems concerning the accuracy of these impressions. Regarding health professions, strong stereotypes exist about medical doctors and nurses. For instance, nurses are often seen as good communicators, nurturing, feminine, and caring [14,15], while doctors are described as confident and decisive [16]. Regarding dentists, it has been documented that stereotypes related to gender, such as the belief that females are more emotionally and relationally competent than males, impact the expectations and impressions of male and female dentists alike. For instance, a study reported that female dentists were expected to spend more time talking to their patients, while male dentists were expected to value patients’ tolerance of pain without complaints [17].

Although logic dictates that stereotypes could be either good or bad, evidence suggests that stereotypes are most often negative. In their review, Baumeister et al [18] argue that illusory correlation appears to form more easily between a social group and negative or bad (distinctive) behaviors compared with positive or neutral behavior, and bad information about a person has more impact on impression formation than good information. It appears to be easier to acquire bad reputations than good reputations because fewer instances of bad behavior are needed to confirm this to be indicating a bad trait or disposition compared with good behavior [18]. Stereotypes or social categories are quite easily learned by social learning processes, but interestingly, stereotypes appear to become more extreme and less variable through social learning processes [19]. Such distortions related to the social learning of stereotypes could then negatively influence people’s thoughts about certain social groups or categories, including professions.

Negative or bad behaviors could be a major influence on professional stereotypes when such behaviors are perceived as distinctive to the profession. In case of dentists and dentistry, bad distinctive behaviors could include instances of painful treatment. For instance, in a study of Norwegian adults, 20%-30% rated their last dental visit as moderately painful or worse and 60% reported having at least one very painful experience at the dentist’s office [20]. Also, a study of Canadian adults found that 42.5% reported having moderate to severe pain during their last dental treatment [21]. In light of these findings, it would be reasonable to assume that painful experiences might serve as a foundation for creating negative stereotypes in relation to dentists. This notion appears to be supported by the findings of Thibodeau and Mentasti [22], who reviewed 100 movies portraying dentists in Western culture. In this study, it was shown that visits to the dentist in movies are often portrayed as a negative and painful experience, where the dentist is being depicted as “…incompetent, menacing, sadistic, immoral, unethical, or corrupt, and one might assume that all dentists behave in this manner.” This association between negative experiences and the public image of dentists has been found in large population studies as well [23], which would not only hamper the image of dentists [23] but also be regarded as a factor in both the maintenance and establishment of dental anxiety [24]. The impact of the negative occupational stereotype related to dentistry could be that people exposed to it are reluctant to seek dental care, and some authors have argued that the dental community should consider promotional campaigns or marketing strategies to dispel the negative images associated with dentistry and to influence reluctant patients [25,26].

Based on the findings that indicate the existence of negative emotions related to dentistry and dentists, we would expect that these associations and stereotypes influence how the profession is talked about in social media. The current study seeks to investigate the language used in Twitter posts about dentists and compare these posts with those about another well-known health profession (medical doctors). We hypothesize that (A) tweets about dentists will have more negative emotion-related words than those about medical doctors and (B) pain-related words are used more frequently in tweets about dentists than in those about medical doctors.

## Methods

### Data Source

Text data were collected from Twitter over a 4-week period starting in the last week of May 2015. For data collection, we used 140Dev server software [27], which ran at a server of the Northern Research Institute in Tromsø, Norway. The server monitored and stored all tweets containing the search terms “dentist” and “doctor”. During the study period, the server downloaded and stored 524,958 tweets containing the word “dentist” and 1,821,914 tweets containing the word “doctor.” To preserve the tweeters’ privacy, none of the supplemental user information available from Twitter was downloaded. The study can, therefore, be said to build only upon nonidentifiable information.

The tweets were in English only, which enabled us to perform the analysis on content written in a single language. Using single, common English words as search terms resulted in over 2.3 million tweets collected over 4 weeks. This design, thus, had the advantage of achieving a large sample size over a relatively short time period.

### Data Selection and Preparation

Because the research questions of the study were related to how most people use Twitter to communicate about health professionals, we made an informal analysis of the suitability of the collected material by browsing through a random selection of tweets from each database. Based on the screening process, we decided that selection criteria would have to be imposed on the material because the text data contained many entries that were outside of the scope of this study (eg, commercial content). In order to remove irrelevant content and to increase the likelihood that selected tweets were personal and relevant to the tweets’ authors, we used personal and possessive pronouns to filter the data. Thus, we excluded all tweets without at least one of the following words present: “I,” “me,” “my,” “mine,” “we,” “us,” “our,” and “ours.” The process was automated using a simple custom-made algorithm ensuring that this selection was case insensitive. A selection of tweets with personal or possessive pronouns included are shown in Textbox 1. In addition, only original tweets were chosen for analysis, that is, tweets tagged as being retweets were excluded.

To obtain approximately the same amount of text data for each target group, we saved a random selection of 10,000 rows of the databases as text files for each target group (eg, one text file containing 10,000 lines for dentists and a similar text file for doctors).

### Data Analysis

To investigate the research hypotheses, we ran these files through Linguistic Inquiry and Word Count (LIWC) [28]. LIWC is a computer application that analyzes text files according to a predefined dictionary and gives information about the percentage of words in the text files that matches the dictionary. The current study used the built-in English language dictionary. Other authors have found LIWC to be a valid approach for measuring emotion in verbal expression [29]. LIWC differs from sentiment programs (such as SentiStrength), which typically give an overall (positive and negative) sentiment [30]. LIWC gives detailed information about the use of different categories of words (ie, “anger,” “anxiety,” and “sadness”), which allows for a lexicologically framed analysis. In order to investigate Hypothesis A, we looked specifically at the word categories in the LIWC dictionary related to negative emotions (Negative emotions and 3 categories of specific negative emotions: Anger, Anxiety, and Sadness) and an overall emotional category (Affective processes) and Positive emotions.

In order to investigate Hypothesis B, we selected synonyms of pain and pain-related words from a popular Web-based English dictionary [31], which were then added to the dictionary of the LIWC analysis software to provide a separate pain category (Textbox 2).

**Figure 1:**
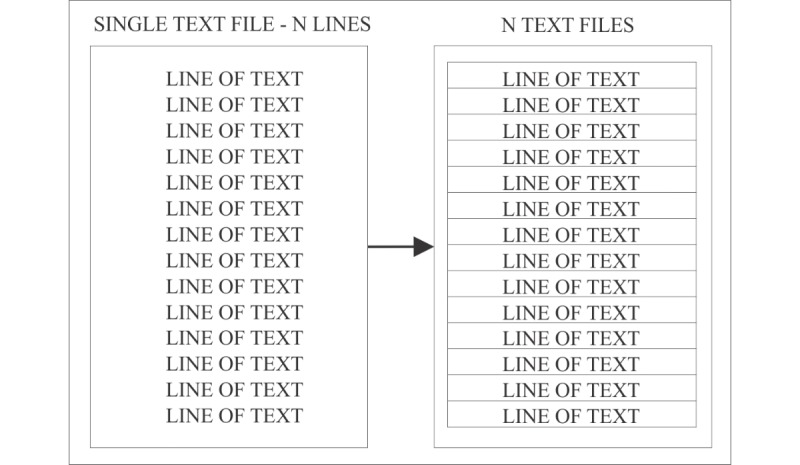
Visualization of a single text file with N lines of text segmented into N text files.

Descriptive analyses were performed with a single file for each target group, while specific hypotheses testing required segmentation of the text files to simulate individual tweets. For the purpose of this study, we used 1000 segments per text file, which was done automatically by choosing this option in LIWC (see Figure 1 for a visualization of the segmentation process). Because the data is not normally distributed, nonparametric Wilcoxon-Mann-Whitney tests were used to test the equality of the distributions. Data were analyzed using Jeffreys’s Amazing Statistics Program (version 0.8.6; JASP Team) [32] and SPSS (version 24; IBM Corp) [33].

**A selection of relevant tweets with pronoun filtering enabled; pronouns in italics.**

**Dentists as the target group:**

*“I* hate the dentist man. Leave *my* wisdom teeth aloneeeee. They not bothering *me”*

“just went to the dentist and *my* mouth feels like someone has punched it”

“Someone come to the dentist with *me. I*'m scared”

**Medical doctors as the target group:**

*“I* tweet this on a daily basis. But *I* truly dislike *my* doctor office.”

*“My* ear is still ringing... Time to go back to the doctor.”

“Hearing the doctor say *I*'m out for 6 weeks is probably the worst thing that has happened to *me.”*

**Synonyms of pain used as a category in the Linguistic Inquiry and Word Count English language dictionary. Superscripted “a” indicates that a word stem was used and all words containing this word stem were counted.**

ache, ached, aches, aching^a^, affliction^a^, agony, burn, burned, burns, burnin^a^, burnt, cramp, cramped, cramps, discomfort^a^, hurt, hurts, hurtful^a^, illness^a^, injur^a^, irritation^a^, maladies, malady, misery, pain, painf^a^, pains, sickness^a^, sore, soreness^a^, sores^a^, sting, stings, stingy, stitch, stitches, strain^a^, suffer^a^, tenderness^a^, throb^a^, throe^a^, tingle^a^, torment^a^, torture^a^, trouble, troubles, troubled, twinge^a^, wound^a^

## Results

The data selected for analyses contained a total of 166,266 words for “dentist” (704,397 characters without spaces) and 182,311 words for “doctor” (776,152 characters without spaces). Table 1 shows the mean proportions of emotional words for each professional category (group). In addition, grand mean baseline values for word categories across different writing tasks [28] have been included for comparison (Table 1).

The analysis (Table 1) showed that there was a difference between tweets about dentists and tweets about doctors, with more affective words used in tweets about dentists (*z*=−6.80; *P*<.001), but a significant difference was not found for positive emotion-related words. Regarding Hypothesis A, the Wilcoxon-Mann-Whitney test showed that more negative words were used in tweets about dentists than in those about medical doctors (*z*=−10.47; *P*<.001). Similarly, tests of equality of distributions were performed for the specific emotion categories. These tests showed a difference in the proportions of words related to anger (*z=*−12.54; *P*<.001), anxiety (*z*=−6.96; *P<*.001), and sadness (*z*=−9.58; *P*<.001), with more words from these categories in tweets about dentists than in those about doctors. Also, Hypothesis B was supported since more pain-related words were used in tweets about dentists than in those about doctors (*z*=−8.02; *P*<.001).

**Table 1:** Mean proportions and SDs of emotional and pain-related word categories, grand mean baseline values from the LIWC documentation, and comparisons of the equality of distributions for the emotional word categories over professional categories.

Word category	Dentist (n=1000), mean (SD)	Doctor (n=1000), mean (SD)	Baseline, mean	*P* value^a^
Affective processes	6.29 (1.87)	5.63 (1.72)	4.41	<.001
Positive emotions	3.22 (1.46)	3.14 (1.43)	2.74	.15
Negative emotions	3.05 (1.41)	2.45 (1.22)	1.63	<.001^b^
Anxiety	0.42 (0.53)	0.34 (0.47)	0.33	<.001^b^
Anger	1.25 (0.92)	0.93 (0.76)	0.47	<.001^b^
Sadness	0.53 (0.56)	0.41 (0.49)	0.37	<.001^b^
Pain	0.34 (0.45)	0.25 (0.40)	N/A^c^	<.001^b^

^a^Wilcoxon-Mann-Whitney test.

^b^The alternative hypothesis specifies that group dentist is greater than group doctor (1-tailed); other tests are 2-tailed.

^c^N/A: not applicable; pain-related words are not included in the original LIWC dictionary.

## Discussion

### Principal Findings

This study demonstrated that more negative emotion words were used in tweets related to dentists than in those related to medical doctors. Thus, compared with medical doctors, dentists seemed to be associated with more negative emotions in tweets. Tweets about dentists did contain more affective words than tweets about medical doctors, but not more positive emotions. This study could be seen as supporting the idea that there is a negative stereotype related to dentists on Twitter. Twitter may, therefore, be one of several channels where the negative stereotype is transmitted, spread, and learned, as other research works indicate that emotions can be spread through both Web-based [34] and real-life social networks [35].

Dental anxiety is a widespread problem [36,37]. The results of the present study can perhaps be seen in unison with the idea that there are several pathways to developing dental anxiety. In a recent qualitative study of Web-based videos related to dental anxiety, 3 main pathways were outlined: direct experiences with aversive dental treatment, vicarious learning through parents and peers, and exposure to negative information [38]. In light of this, negative occupational stereotyping might be an important factor in the development of dental anxiety, as it creates negative associations and expectations irrespective of the individuals’ own experiences. Thus, ambiguous stimuli or information in the dental situation might be interpreted negatively based on the negative emotions and expectations related to the stereotype. For instance, nonverbal communication such as the tone of voice used by the dentist when providing information might be considered as condescending or authoritative by some patients due to negative expectations, while patients without negative expectations are less prone to drawing similar conclusions.

This study supported the hypothesis that negative emotions would be more frequently used in relation to dentists than to doctors. For anxiety words, this was expected given that a potential occupational stereotype related to dentistry can be linked to the relatively widespread phenomena of subclinical dental anxiety or low-grade or moderate worry about dental treatment, which is believed to be quite prevalent in most societies [39,40]. More surprising, perhaps, was the differences observed for both anger and sadness, which can be more difficult to understand. However, it is a quite common finding that people are willing to share anger or anger-related materials on the Web [41] and that feelings of anger might be related to the idea that dental treatment is somehow unethical in the sense that it is expensive, painful, or administered without proper consent [42,43]. Also, the motivational direction of anger is argued to be different from some other feelings in that anger is an approach-oriented emotion concerned with removal of an obstacle rather than withdrawal or avoidance from the obstacle [44,45]. Thus, people might be motivated to write about (ie, approach) their angry feelings about dentists and dental treatment. This is, in part, supported by the fact that anger words are used more than other specific negative emotional words in this study’s data. It is also noteworthy that positive words were more frequent than negative words for both doctors and dentists; however, the existence of positive information or information that disconfirms stereotypes is often not effective in hindering the spread of stereotypes [46].

In addition, we found an expected difference between tweets concerning dentists and medical doctors for pain-related words, with more pain-related words used in relation to dentists. This might be a testament to the significance of pain in relation to dentistry [20,21], but it poses the question why pain is not as significant in relation to medical doctors. For instance, a visit to the doctor might very well be associated with pain and discomfort. A possible reason for the current results might be how pain is perceived in these different contexts. Pain related to a health problem is most often alleviated through interaction with a medical professional (ie, a doctor) either through a medical procedure or prescribed painkillers (eg, a visit to our general practitioner for help with acute back pain or a swollen knee after a fall). This also applies to acute dental problems. However, in the case of nonacute dentistry, it might be argued that pain is caused by the visit to the dentist rather than be a byproduct of necessary examinations or treatment. This might happen because we are expected to get frequent dental check-ups to prevent dental problems even though we are symptom free [47], while the notion of preventive medical check-ups appears to be related strongly to the concept of explicit risk factors [48]. Thus, we might end up receiving painful dental treatment and suffering both physical and financial discomforts for which there is no apparent reason (to the layperson), except for the professional opinion of the dentist. Such differences in the perception of pain related to these professions might provide us with some explanation for the differences observed in the current study, and it is important to consider pain experiences in the dental setting as a key factor in determining patient satisfaction [49,50].

### Limitations

As is often the case with studies of language in natural settings, the results of this study will have to be viewed in light of the inherent challenges in interpreting language and language elements (eg, manifest content) in relation to social or psychological processes (eg, latent content). Specifically, we propose that more negative words in tweets about dentists are related to the existence of a negative occupational stereotype or negative expectations related to dentists. These findings might influence, or be a reflection of, people’s behaviors, beliefs, or attitudes related to oral health. How differences in word categories influence real-life learning processes or reasoning, as suggested here, is not clear. However, the relevance of investigating linguistic data and word counts in relation to thinking and behavior has been demonstrated elsewhere for a wide range of issues [51-54]. While our study results support the existence of a negative occupational stereotype and negative expectations related to dentists, as others have argued previously [22,26,55], the actual impact of the stereotypes and expectations are outside the scope of this study. Also, the specificity of the search terms and single language content will impact the generalizability of the current results. In future studies, longer study periods, inclusion of more search terms, and a deeper look into ancillary data (ie, retweets and likes of tweets) could give larger sample sizes and additional insights.

### Conclusions

In conclusion, our study suggests that dentists are tweeted about in more negative terms than medical doctors. More research is needed concerning the potential impact of this on dental patients’ health-related behavior and beliefs. It is unclear what can be done to reduce the proportion of dentist-related tweets with negative emotion-related words or the potential impact of a negative occupational stereotype about dentists expressed in social media. Potential interventions, however, could include informational campaigns on social media that could underline positive aspects of dental health and dentistry [56,57], interventions highlighting preventive dental care [25], interventions aimed at reducing both actual dental costs and uncertainty about dental cost [23,58], and increasing focus on the importance of provider-patient interaction in dental education [59].

## Abbreviations

LIWC: Linguistic Inquiry and Word Count
